# Metabolite marker discovery for the detection of bladder cancer by comparative metabolomics

**DOI:** 10.18632/oncotarget.16393

**Published:** 2017-03-21

**Authors:** Chi-Hung Shao, Chien-Lun Chen, Jia-You Lin, Chao-Jung Chen, Shu-Hsuan Fu, Yi-Ting Chen, Yu-Sun Chang, Jau-Song Yu, Ke-Hung Tsui, Chiun-Gung Juo, Kun-Pin Wu

**Affiliations:** ^1^ Institute of Biomedical Informatics, National Yang Ming University, Taipei 11221, Taiwan; ^2^ Department of Urology, Chang Gung Memorial Hospital, Taoyuan 33305, Taiwan; ^3^ College of Medicine, Chang Gung University, Taoyuan 33302, Taiwan; ^4^ Department of Biomedical Engineering, National Yang Ming University, Taipei 11221, Taiwan; ^5^ Proteomics Core Laboratory, China Medical University Hospital, Taichung 40402, Taiwan; ^6^ Department of Medical Research, China Medical University Hospital, Taichung 40402, Taiwan; ^7^ Graduate Institute of Integrated Medicine, China Medical University, Taichung 40402, Taiwan; ^8^ Molecular Medicine Research Center, Chang Gung University, Taoyuan 33302, Taiwan; ^9^ Department of Biomedical Sciences, Chang Gung University, Taoyuan 33302, Taiwan; ^10^ Department of Cell and Molecular Biology, Chang Gung University, Taoyuan 33302, Taiwan

**Keywords:** bladder cancer, metabolomics, metabolite marker selection, decision tree, machine learning

## Abstract

Bladder cancer is one of the most common urinary tract carcinomas in the world. Urine metabolomics is a promising approach for bladder cancer detection and marker discovery since urine is in direct contact with bladder epithelia cells; metabolites released from bladder cancer cells may be enriched in urine samples. In this study, we applied ultra-performance liquid chromatography time-of-flight mass spectrometry to profile metabolite profiles of 87 samples from bladder cancer patients and 65 samples from hernia patients. An OPLS-DA classification revealed that bladder cancer samples can be discriminated from hernia samples based on the profiles. A marker discovery pipeline selected six putative markers from the metabolomic profiles. An LLE clustering demonstrated the discriminative power of the chosen marker candidates. Two of the six markers were identified as imidazoleacetic acid whose relation to bladder cancer has certain degree of supporting evidence. A machine learning model, decision trees, was built based on the metabolomic profiles and the six marker candidates. The decision tree obtained an accuracy of 76.60%, a sensitivity of 71.88%, and a specificity of 86.67% from an independent test.

## INTRODUCTION

Bladder cancer (BCa) is the ninth most common cancer in the world; 429,000 new cases and 165,000 deaths were estimated in 2012 [1]. According to the most recent estimates of the American Cancer Society, in 2016 there will be 76,960 new cases of BCa and 16,390 deaths from BCa annually in the United States [2]. A 2016 official report of the Taiwan government said that in Taiwan there were 2,055 new cases of BCa (accounting for 2.07% of all cancers) and 833 deaths (1.86% of all cancers) in 2013 [3]. Currently, cystoscopy and cytology are standards for BCa detection. Cystoscopy is an invasive, annoying and costly procedure, and may fail to exam certain areas in bladder [4]. Cytology is a noninvasive method and often serves as an adjunct to a negative cystoscopy study. Although cytology has high specificity, its sensitivity is not satisfactory, particularly for low-grade tumors [5]. Identifying discriminative markers for the noninvasive detection of BCa is therefore essential. Several candidate protein markers for BCa have been identified from urine and bladder cancer cells; these markers are used for the initial diagnosis and monitoring recurrence and treatment response. Nevertheless, the sensitivity and specificity of these markers are not superior to existing detection methods, and the clinical utility of these markers has not been comprehensively examined [6–9]. Thus, there is a compelling need to develop more reliable BCa markers.

One promising approach to the BCa detection and marker discovery is to study the urine metabolome during the occurrence of the disease. Urine is in direct contact with bladder epithelia cells that may give rise to BCa; metabolites released from bladder cancer cells may be enriched in urine samples. Urine metabolomics have been used in BCa detection and marker discovery. Both mass spectrometry (MS) and nuclear magnetic resonance (NMR) spectroscopy have been applied to identify changes in expression level of urinary metabolites [10]. Srivastava et al. [11] used ^1^H NMR spectroscopy to perform urine metabolomic profiling against 103 BCa patients and controls. They found that the concentration of taurine in urine was significantly elevated in BCa samples, and therefore suggested taurine as a putative marker. Issaq et al. [12] performed urine metabolomic profiling against 48 healthy individuals and 41 patients with bladder transitional cell carcinoma by liquid chromatography/mass spectrometry (LC/MS). The metabolomic profiles were subjected to the orthogonal projection to latent structures-discriminant analysis (OPLS-DA) and principal component analysis (PCA). The results of OPLS-DA and PCA showed a clear separation between patient and control profiles. Pasikanti et al. [13] applied gas chromatography/mass spectrometry (GC/MS) to profile urine metabolites of 24 BCa and 51 non-BCa samples. They selected 11 putative markers that were related to glycolysis. Jin et al. [14] applied LC/MS to profile metabolites of 138 patients with BCa and 121 control subjects. The study identified 12 putative markers that were involved in glycolysis and betaoxidation. Their multivariate regression analysis also suggested that the metabolomic profiles may correlate with survival time. Peng et al. [15] developed a quantitative approach, universal metabolome-standard (UMS), in conjunction with LC/MS to perform metabolomic profiling. The platform was used for marker discovery on 91 BCa patients and 99 control subjects. They reported 10 putative markers, and some of the markers were involved in phospholipid metabolism and glycolysis. Shen et al. [16] used LC/MS to perform metabolomic profiling against 23 patients with early stage BCa and 21 healthy controls. They identified six putative markers GlyCysAlaLys, nicotinuric acid, AspAspGlyTrp, inosinic acid, trehalose, and ureidosuccinic acid. Wittmann et al. [17] applied LC/MS to profile metabolites of 66 BCa and 266 non-BCa subjects. They identified palmitoyl sphingomyelin, lactate, adenosine, succinate, and arachidonate as putative markers. The authors also suggested that metabolites related to lipid metabolism may be potential BCa markers. Although there have been several studies for BCa detection and marker discovery based on urine metabolome, further urine metabolomic profiling may still yield new putative markers due to the variable, dynamic, and diverse nature of urine metabolomes.

In this study, we applied ultra-performance liquid chromatography time-of-flight mass spectrometry (UPLC-TOF-MS) to perform metabolomic profiling on 87 samples of BCa patients and 65 samples of hernia patients. Statistical analysis and cross validation revealed that machine learning models built on metabolomic profiles can discriminate BCa samples from hernia samples. There were six spectral ions selected as putative BCa markers. Two of the marker ions were identified as imidazoleacetic acid. The sources of imidazoleacetic acid, histidine and histamine, have been reported in connection with BCa. The result suggests that imidazoleacetic acid has the potential to be a BCa marker.

## RESULTS

### Subject characteristics

There were totally 152 enrolled subjects, in which 87 were diagnosed with BCa and 65 diagnosed with hernia. Hernia patients served as controls in this study. The demographics of enrolled subjects were summarized in Table 1. The BCa patients comprised 54 males and 33 females and had an average age of 68.2±14.5. The controls comprised 62 males and 3 females and had an average age of 64.6±13.2. Creatinine, an important index in urine test, was statistically at the same level in BCa patients and controls (p value = 0.203). However, Hemoglobin, another important index in urine test, was statistically lower in the BCa patients than in the controls (p value < 0.001) as hematuria being the common finding in BCa. Within all BCa patients, 55 were diagnosed with early stage BCa tumor and 32 were diagnosed with advanced stage BCa tumor. The early stage BCa tumor denotes the superficial tumor without muscle involvement, while the advanced stage BCa tumor denotes the tumor invading to muscle layer. The 152 subjects were randomly partitioned into two sets, training set and testing set. The training set was used to select metabolite markers and build a predictive model for BCa; the training set contained 105 subjects, including 55 BCa patients and 50 hernia patients. The testing set was used to evaluate the performance of the predictive model built using the training set; the testing set contained 47 subjects, including 32 BCa patients and 15 hernia patients.

**Table 1:** Patient characteristics

	BCa	Hernia	p value
**# of subjects**	87	65	
**Age ± SD**	68.2±14.5	64.6±13.2	0.117
**Gender**			
Male	54 (62%)	62 (95%)	
Female	33 (38%)	3 (5%)	
**Creatinine**	1.40	1.11	0.203
**Hemoglobin**	12.33	13.67	< 0.001*
**Tumor stage**			
Early^1^	55		
Advanced^2^	32		

* Statistically significance, as hematuria being the common finding in BCa

1 Early stage: superficial tumor without muscle involvement

2 Adcanced stage: tumor invasion to muscle layer

### Metabolomic profiling of BCa and hernia urine samples

**Figure 1:**
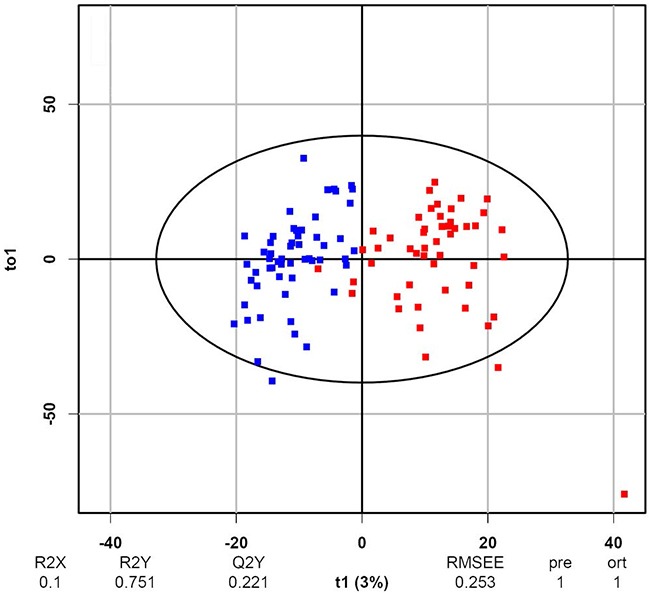
OPLS-DA score plot of the BCa and hernia metabolomic profiles Each box represents the metabolomic profile of 944219 spectral ions of an individual subject. There are 87 blue boxes representing BCa patients and 65 red boxes representing hernia patients.

The urine samples of the 152 enrolled patients were subjected to the UPLC-TOF-MS analysis for metabolomic profiling, and 944219 spectral ions were identified for each sample (see Supplementary Tables 1, 2, 3, 4 for BCa samples and Supplementary Tables 5, 6, 7 for hernia samples). To test whether or not UPLC-TOF-MS-based metabolomic profiling be an effective approach to discriminate BCa samples from hernia samples, we used the metabolomic profiles of the training set to construct an OPLS-DA [18] model with one predictive component and two orthogonal components. The obtained OPLS-DA score plot was depicted in Figure 1. The satisfactory separation (R^2^X_cum_ = 0.1, R^2^Y_cum_ = 0.751, Q^2^_cum_ = 0.221) between BCa and hernia samples in the plot showed the discriminative potential of metabolomic profiling in BCa detection.

**Table 2:** Candidate ions for BCa detection

Candidate ions	# BCa	# Hernia	Ratio	p value	AUC
2.56 min: 314.085 m/z	46	25	1.27	2.62E-05	0.73
3.65 min: 165.007 m/z	38	21	2.24	5.14E-05	0.72
3.65 min: 183.018 m/z	39	23	2.85	7.32E-05	0.72
12.53 min: 194.117 m/z	42	22	11.20	8.15E-05	0.72
19.42 min: 213.146 m/z	55	50	1.47	2.52E-04	0.71
2.04 min: 106.950 m/z	50	36	1.41	4.06E-04	0.70

# BCa: Number of BCa samples containing the ion

# Hernia: Number of hernia samples containing the ion

Ratio: expression fold change of ions in BCa samples to hernia samples

p value: received from Wilcoxon rank sum test

AUC: area under the receiver operating characteristic curve

**Table 3:** Performance of decision trees reported by the 5-fold cross validation

Iteration	Accuracy	Sensitivity	Specificity
1	85.71%	81.82%	90.00%
2	82.14%	79.55%	85.00%
3	86.90%	84.09%	90.00%
4	83.33%	81.82%	85.00%
5	85.71%	81.82%	90.00%
**Average**	84.76% ± 1.75%	81.82% ± 1.61%	88.00% ± 2.74%

Accuracy–the probability that a sample is correctly classified; Sensitivity–the probability that a BCa sample is correctly classified as BCa; Specificity–the probability that a hernia sample is correctly classified as hernia.

### Identification of candidate markers for BCa detection

Since metabolomic profiling revealed a certain degree of discriminative power in BCa detection, we would like to further select discriminative markers from metabolomic profiles. We applied a screening pipeline to perform the marker selection. The pipeline consisted of the following four steps.

1. *Selection by detection count*. An ion was considered as a marker candidate only if it had a nonzero intensity in more than half of the training samples.

2. *Selection by fold change*. The fold changes of all training samples were first subjected to a log transformation and then underwent a fitting of Gaussian distribution. Ions with positive log ratios and located beyond one standard deviation from the mean of the distribution were regarded as significantly up-regulated and chosen as marker candidates. Down-regulated ions were not taken into consideration because we may not sure whether a metabolite was not expressed or our instrument missed detecting it.

3. *Selection by statistical test*. We applied Wilcoxon rank sum test [19] to assess the discriminative power of ions; ions with p value < 0.05 were selected as marker candidates.

4. *Selection by the area under the receiver operating characteristic curve*. Ions with an area under the receiver operating characteristic curve (AUC [20]) ≥ 0.7 were selected as marker candidates.

**Figure 2:**
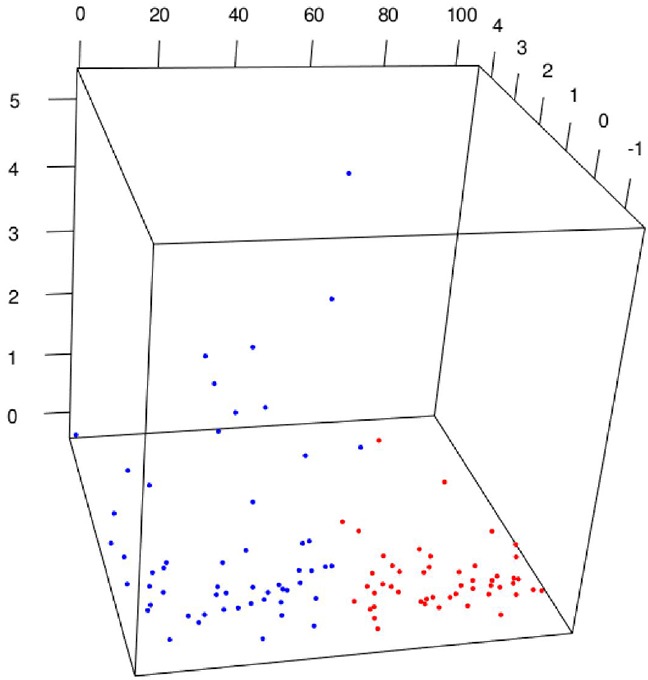
LLE plot of the BCa and hernia profiles of marker candidates Each dot represents the profile of six marker candidates of an individual subject. There are 87 blue dots representing BCa patients and 65 red dots representing hernia patients.

The screening pipeline selected six candidate ions from 944219 spectral ions (Table 2). The training set accordingly underwent a locally linear embedding (LLE) clustering [21] based on the six candidates to validate the discriminative power of the selected markers. The clustering result was shown in Figure 2, which revealed a good separation between BCa and hernia samples.

### Performance of the BCa detection model

**Figure 3:**
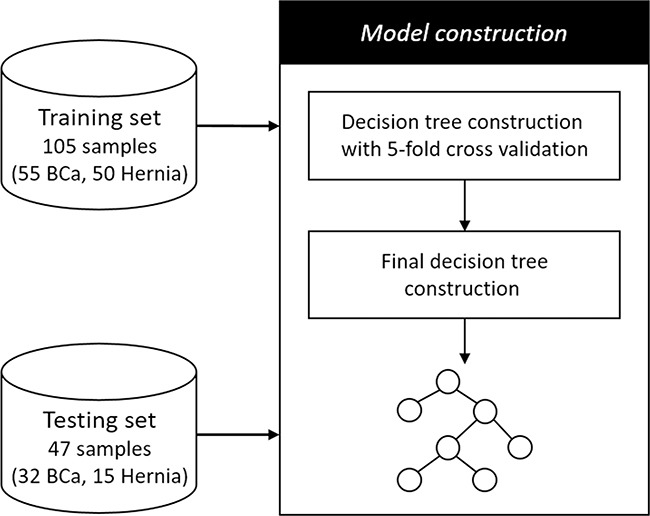
The decision tree construction and evaluation workflow First, the training set of 55 BCa and 50 hernia samples was subjected to a procedure of decision tree construction with 5-fold cross validation to evaluate the stability and generalization of the decision tree model. Second, the whole training set was used to build a final decision tree. Finally, an independent test was performed on the testing set of 32 BCa and 15 hernia samples to validate the final decision tree.

On the basis of the six selected marker candidates, we constructed a predictive model, decision tree, for the detection of BCa. The workflow of the detection model construction was depicted in Figure 3. Each sample was characterized by the six candidates only. To limit overfitting, the training set of 105 samples was first subjected to a 5-fold cross validation to evaluate the stability and generalization of decision tree model. Following the cross validation, a decision tree was constructed using the whole training set. We finally conducted an independent test to evaluate the performance of the constructed decision tree using the testing set of 47 samples.

In the 5-fold cross validation, the training set was randomly partitioned into 5 folds of equal size; each fold contained 21 samples, including 11 BCa samples and 10 hernia samples. In the cross validation, each fold was in turn used for decision tree evaluation and the remaining 4 folds were used for decision tree construction. The evaluation results of the 5-fold cross validation were listed in Table 3. The average accuracy of the cross validation was 84.76% with 1.75% standard deviation, which showed a stable performance of the decision tree approach. The cross validation also reported a stable sensitivity (81.82% ± 1.61%) and specificity (88.00% ± 2.74%). When the final decision tree was evaluated by the testing set of 47 samples, the predictive model received an accuracy of 76.60%, a sensitivity of 71.88%, and a specificity of 86.67%, respectively.

## DISCUSSION

High-throughput chemical analysis techniques, such as MS and NMR, have made urine metabolomics a quick and simple alternative to BCa detection and biomarker discovery [11–17]. In this study, we also found six ion candidates (2.56 min: 314.085 m/z), (3.65 min: 165.007 m/z), (3.65 min: 183.018 m/z), (15.23 min: 323.056 m/z), (19.42 min: 213.146 m/z), and (2.04 min: 106.950 m/z). Only ions (3.65 min: 165.007 m/z) and (3.65 min: 183.018 m/z) were identified as imidazoleacetic acid (Supplementary Figures 1 and 2), and others were unknown metabolites. It is noteworthy that although studies reported in [14–17] as well as ours all targeted on urine metabolome, the signatures identified by these studies were quite different from each other. The difference may originate from various factors. First, there is no standard of sample acquisition, including patient characteristics (age, gender), cancer characteristics (histological type, advancement), and time of collection (before or after surgery) etc. Second, different platforms were used to profile urine metabolites. NMR, LC/MS, and GC/MS can have different separation techniques, chromatographic modes, and mass accuracy and resolution. Third, the environmental stress and food intake greatly impact on the composition of metabolome. These factors all increased the diversity of metabolomic profiles obtained from different laboratories. Most importantly, statistical approaches are very sensitive to data, different control groups might lead to different results. Jin et al. [14] used healthy individuals and patients with hematuria as control subjects. Peng et al. [15] used hernia, urinary tract infection, and hematuria patients as control subjects. Wittmann et al. [17] used hematuria patients, individuals with a history of BCa but no current disease, and healthy individuals as control subjects. Shen et al. [16] used healthy individuals as control subjects. In this study, we used hernia patients as control groups. All these studies used different configurations of control groups, and reasonably identified different metabolite signatures for BCa.

Our only identified metabolite, imidazoleacetic acid, derives from the oxidation of histamine [22]. Mast cells have been reported to be associated with bladder carcinoma [23]. Moreover, the overexpression of cyclooxygenase-2 driven by keratin 5 promoter causes spontaneous inflammation and is related to hyperplasia and carcinomas in urinary bladders [24]. Histamine, primarily released by mast cells in inflammatory processes, may therefore be a potential marker of BCa [25]. Histamine is derived from the decarboxylation of histidine by histidine decarboxylase (HDC) [26]. HDC has been reported to expressed in melanoma [27] and human small cell lung carcinoma [28]. Histidine has been identified as potential marker for BCa [29]; Putluri et al. [30] compared the metabolomic profiles of normal, benign adjacent, and cancerous bladder tissues, and found that histidine was increased in bladder tumors relative to benign adjacent tissues. Alberice et al. [31] further extended the knowledge regarding relevance of histidine with respect to the progression of BCa. Imidazoleacetic acid was reasonably enriched in our BCa metabolome since the overexpression of its sources, histidine and histamine, has been shown in connection with BCa.

## MATERIALS AND METHODS

### Chemicals

All chemicals and solvents were purchased from Sigma–Aldrich (St. Louis, MO, USA). The chemicals were all analytical grade. Water, acetonitrile containing 0.1% formic acid and water containing 0.1% formic acid, were of CHROMASOLV grade.

### Sample preparation

All urine samples were collected at Chang Gung Memorial Hospital, Taoyuan, Taiwan. The study protocol was approved by the Medical Ethics and Human Clinical Trial Committee at Chang Gung Memorial Hospital (IRB approval number 103-3878B). A total of 87 BCa patients containing either non-muscle invasive or muscle invasive diseases were recruited in this study. Additionally, 65 hernia patients were recruited as controls from cancer patients with comparable age and exactly the same procedures of urine sample collection in the first morning after admission before surgical intervention. The diagnosis of BCa was all pathologically proven of urothelial carcinoma after transurethral biopsy or resection of tumor. The urine will discard and exclude for further analysis if the diagnosis was not confirmed. If muscle invasion was identified, radical surgery to remove entire bladder would be suggested but not always be performed according to the decision of patient and family. In the control cohort, all hernia patients were checked for any previous cancer history and would be excluded if positive past history. Cells and debris were removed by centrifugation (5,000 × *g* for 30 min at 4°C). This was done within one hour after sample collection [32]. The sample was then kept at −80°C for long-term storage. Before mass spectrometry analysis, the sample was thawed on 4°C. Freezing-point depression was measured to determine osmolalities of samples using an Advanced Instruments Osmometer Model 3320 (Norwood, MA). All the samples were normalized by diluting their osmolalities to 250 mOsm/kg. 50 μL of urine was diluted with 200 μL of methanol and centrifuged at 13,200 × *g* for 15 min at 4°C. The supernatant was dried using N_2_. The sample was re-dissolved with 50 μL solvent consisting of MeOH: H_2_O (2:1 v/v) and centrifuged at 13,200 × *g* for 15 min at 4°C; the supernatant from this centrifugation was used directly for LC-MS analysis. Equal amount of urine from each sample in the analysis were mixed as the quality control (QC) sample [33].

### Metabolite identification and quantitation by mass spectrometry

All samples were analyzed by using an UPLC-TOF-MS system for further identification and quantitation of metabolites. The UHPLC system (Ultimate 3000; Dionex, Germany) equipped with a C18 reversed-phase column (2.1 × 100 mm, 1.8 μm, HSS-T3; Waters, Milford, MA, USA) was coupled with a hybrid Q-TOF mass spectrometer (maXis impact, Bruker Daltonics, Bremen, Germany) with an orthogonal electrospray ionization (ESI) source. The product ion spectra were acquired by either an ion trap MS (HCT ultra, Bruker Daltonics, Bremen, Germany) or an LTQ-Orbitrap XL (Thermo Scientific, San Jose, CA, USA). The selection of mass spectrometer was depending on the abundance of the ion itself; the ions with high abundances were acquired by the ion trap MS, and the ions with low abundances that cannot be detected by ion trap MS were acquired by LTQ-Orbitrap XL MS. The gradient of LC was that the initial flow rate was 0.1 mL/min of 99% solvent A (0.1% formic acid) and 1% solvent B (acetonitrile with 0.1% formic acid). A volume of 1 μL of sample was injected. After injection, solvent B was maintained at 1% for 5 min, then increased to 50% during a span of 9 min, then to 90% over 6 min, and finally to 99% over a period of 12 min after which this percentage composition was held for 1 min. The flow rate was changed to 0.5 mL/min, and after 5 min reduced to 0.1mL/min. After 0.1 min, solvent B was reduced back down to 1% and held at this percentage for 7 min.

The Q-TOF mass spectrometer was operated in positive ion mode using the m/z range 50–1000 at 1 Hz (summation value of 9839) for urine screening. The capillary voltage of the ion source was set at +3300 V, and the endplate offset was 500 V. The nebulizer gas flow was 1 bar and drying gas flow was 8 L/min. The drying temperature was set at 200°C. The radio frequencies (RF) of Funnel 1 and Funnel 2 were both 100 Vpp. The hexapole RF was 120 Vpp and the low mass cutoff of quadrupole was 30 m/z. The product ion spectra were all acquired with the default setting of mass spectrometer. Instrument calibration was performed externally prior to each batch run with 1 mM sodium formate solution in isopropanol/water (9:1, v/v). The spectra from 30 min to 32 min of each LC/MS analysis were the sodium formate clusters; these spectra were averaged for calibration [34]. The spectra of each run were calibrated automatically by using Profile Analysis 2.0 (Bruker Daltonics, Bremen, Germany), and high-precision calibration method was applied for the instrument calibration. Before batch analysis, the QC sample was injected 10 times to condition the UPLC column. The sample injection sequence was randomized, according to the suggestions of Want et al., to reduce the effect of contamination from the previous injection(s) [33]. After every 10 urine sample analyses, the QC sample was injected to check the stability of the system through the whole analysis. Each identified spectral ion was denoted by (*x* min: *y* m/z) and implicitly with its intensity, where *x* and *y* were the retention time and m/z of the ion, respectively. All identified ions in a spectrum formed a metabolomic profile of a sample and were subjected to the following marker selection pipeline. The selected ions were regarded as marker candidates and searched against databases Metlin and HMDB for metabolite identification [35–37]. Marker candidates were further confirmed by interpreting their product ion spectra and/or matching with the retention time and exact masses of authentic standards.

### Bioinformatics and statistical analysis

The supervised discriminant analysis OPLS-DA and the unsupervised learning approach LLE were used to measure the degree of separation between metabolomic profiles of BCa and hernia samples. Three-parameter Gaussian fitting, Wilcoxon rank sum test, and AUC were used to select significantly up-regulated marker candidates. In three-parameter Gaussian fitting, we tried to find the mean, amplitude, and standard deviation that best described our log ratios as a Gaussian distribution. The decision tree algorithm C4.5 was used to construct our BCa detection models. C4.5 algorithm builds a decision tree by calculating the gain ratio of features from training data [38]. In this study, R version 3.3.2 [39] was used to perform OPLS-DA, LLE, Wilcoxon rank sum test, and AUC calculation. J48 program in the WEKA data mining toolkit was used to build our decision tree; J48 is an open source Java implementation of the C4.5 algorithm [40]. The accuracy, sensitivity, and specificity were used to evaluate the performance of our decision trees. The accuracy is the probability that a sample is correctly predicted. The sensitivity is the probability that a BCa sample is correctly predicted as BCa. The specificity is the probability that a hernia sample is correctly predicted as hernia.

## SUPPLEMENTARY MATERIALS FIGURES AND TABLES
















