# Supplementary material for: Metabolite marker discovery for the detection of bladder cancer by comparative metabolomics
Source: Oncotarget. 2017 Mar 21;8(24):38802–10. doi: 10.18632/oncotarget.16393 (PMC5503573; doi:10.18632/oncotarget.16393)
Supplement: Supplementary file 1 [file oncotarget-08-38802-s001.pdf]

Metabolite marker discovery for the detection of bladder cancer by comparative metabolomics

SUPPLEMENTARY FIGURES AND TABLES

The product ion spectra of imidazoleacetic acid. In the following figures, imidazoleacetic acid was confirmed by its authentic standards.

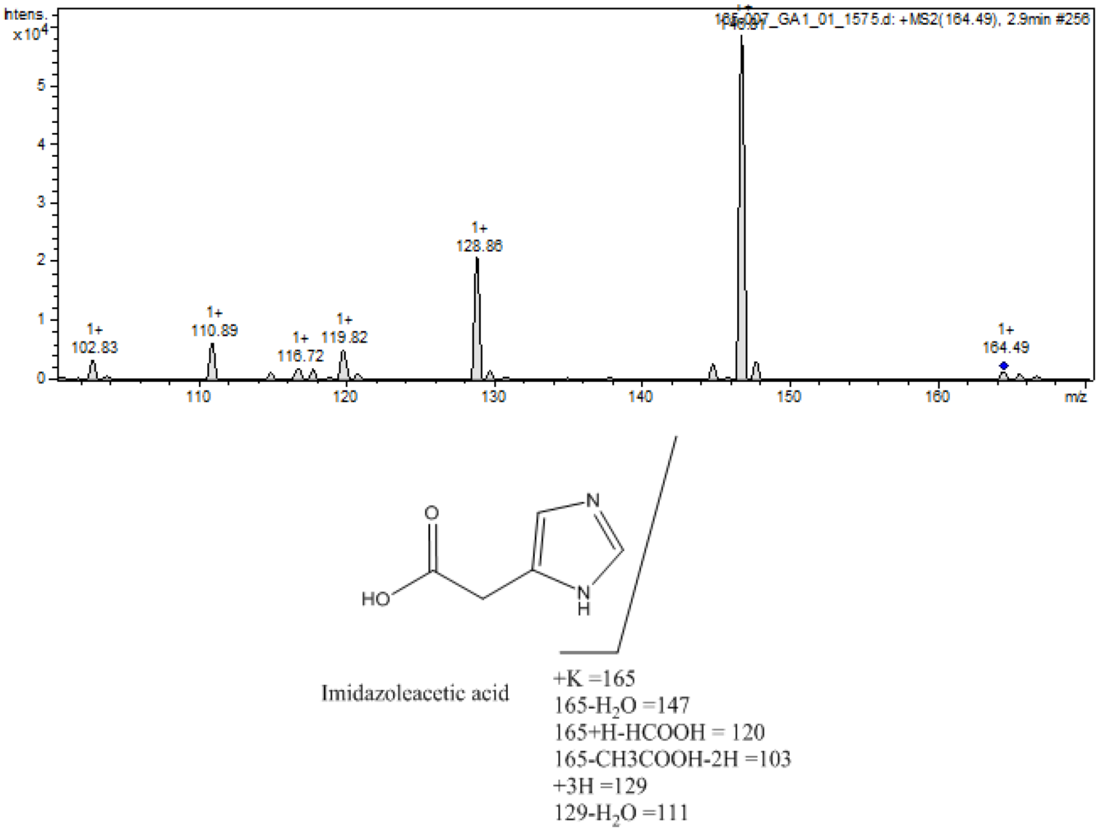

Supplementary Figure 1: The product ion spectra of 3.65min:165.007m/z.

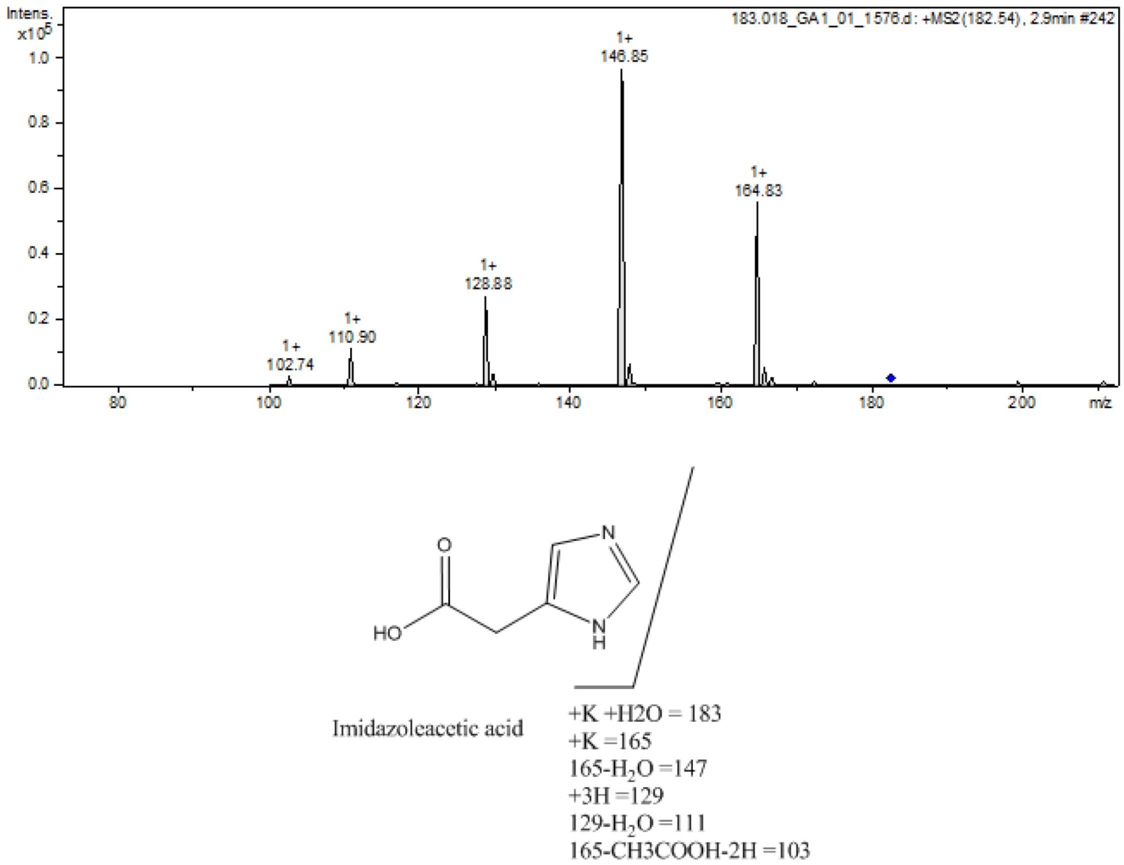

Supplementary Figure 2: The product ion spectra of 3.65min:183.018m/z.

Supplementary Table 1: The spectral raw data of bladder cancer patients #1 to #25.

See Supplemenatry File 1

Supplementary Table 2: The spectral raw data of bladder cancer patients #26 to #50.

See Supplemenatry File 2

Supplementary Table 3: The spectral raw data of bladder cancer patients #51 to #75.

See Supplementary File 3

Supplementary Table 4: The spectral raw data of bladder cancer patients #76 to #87.

See Supplementary File 4

Supplementary Table 5: The spectral raw data of hernia patients #1 to #25.

See Supplementary File 5

Supplementary Table 6: The spectral raw data of hernia patients #26 to #50.

See Supplementary File 6

Supplementary Table 7: The spectral raw data of hernia patients #51 to #65.

See Supplementary File 7
